# Seroprevalence of *Toxoplasma gondii *infection in dairy cattle in southern China

**DOI:** 10.1186/1756-3305-5-48

**Published:** 2012-03-09

**Authors:** Dong-Hui Zhou, Fu-Rong Zhao, Ping Lu, Hui-Yan Xia, Min-Jun Xu, Li-Guo Yuan, Chao Yan, Si-Yang Huang, Shou-Jun Li, Xing-Quan Zhu

**Affiliations:** 1 State Key Laboratory of Veterinary Etiological Biology, Key Laboratory of Veterinary Parasitology of Gansu Province, Lanzhou Veterinary Research Institute, Chinese Academy of Agricultural Sciences, Lanzhou, Gansu Province 730046, People's Republic of China; 2 College of Veterinary Medicine, South China Agricultural University, Guangzhou, Guangdong Province 510642, People's Republic of China; 3 China Animal Health and Epidemiology Center, Qingdao, Shandong Province 266032, People's Republic of China; 4 Department of Pathogen Biology and Immunology, Xuzhou Medical College, Xuzhou, Jiangsu Province 221004, People's Republic of China; 5 College of Animal Science and Technology, Yunnan Agricultural University, Kunming, Yunnan Province 650201, People's Republic of China; 6 College of Animal Science and Veterinary Medicine, Heilongjiang Bayi Agricultural University, Daqing, Heilongjiang Province 163319, People's Republic of China

## Abstract

**Background:**

As an obligate intracellular parasite, *Toxoplasma gondii *can infect humans and almost all warm-blooded animals. The consumption of raw or undercooked beef and milk is considered a risk for *T. gondii *infection in humans. However, little is known of *T. gondii *infection in dairy cattle in metropolitan Guangzhou, southern China. This study was performed to determine the seroprevalence of *T. gondii *in dairy cattle in Guangzhou, southern China.

**Findings:**

Serum samples were collected from 350 dairy cattle on five farms in Guangzhou, China from 2009 to 2010, and all of the 350 serum samples were examined for specific antibodies to *T*. *gondii *by indirect hemagglutination antibody test (IHA). The overall seroprevalence of *T. gondii *in dairy cattle was 5.7% (20/350). Among these examined dairy cattle, dairy cattle which were < 6 year old or ≥ 5 year old had the highest seroprevalence of 12.5% followed by those dairy cattle which were < 5 year old or ≥ 4 year old (8%); dairy cattle with 3 pregnancies had the highest seroprevalence (11.5%), among the examined dairy cattle, although these differences were not statistically significant.

**Conclusions:**

The results of the present survey indicate that *T. gondii *infection is prevalent in dairy cattle of all age ranges in Guangzhou, southern China, which may be a risk factor for human infection with *T. gondii *in this region.

Dong-Hui Zhou and Fu-Rong Zhao contributed equally.

## Background

*Toxoplasma gondii *is an obligate intracellular protozoan parasite, infecting humans and almost all warm-blooded animals and causing serious zoonotic toxoplasmosis, with a worldwide distribution [1-5]. Toxoplasmosis is an important food-borne parasitic disease, which is usually asymptomatic in immunocompetent individuals but can cause toxoplasmic encephalitis in immuno-compromised patients, blindness, abortion, fetal abnormalities or even prenatal death in congenital cases [1-3]. Humans and animals acquire infection mainly by the consumption of raw and undercooked meat, and also by the ingestion of *T. gondii *oocysts present in the environment (water, soil, fruits and vegetables) contaminated with the faeces of infected cats [1-3].

Although being considered a poor host for *T. gondii*, both natural and experimental infections of *T. gondii *in cattle have been reported [1,6]. Infection with the parasite may cause abortion, resulting in substantial economic loss and also the potential to transmit to other animals and humans [7]. Studies have indicated that the consumption of raw or undercooked beef and milk may be a risk for *T. gondii *infection in humans [8].

*T. gondii *seroprevalence has been documented in humans, cats, dogs, rats, ducks and chickens in Guangzhou, southern China [9-13]. However, little is known of the infection of *T*. *gondii *in dairy cattle in this city. The objective of the present investigation was to determine *T. gondii *seroprevalence in dairy cattle from dairy farms in Guangzhou, southern China by using an indirect hemagglutination antibody test (IHA). The results of the survey will provide base-line data for the implementation of effective strategies and measures for the control and prevention of *T. gondii *infection in dairy cattle in this southern city.

## Methods

### Serum preparation

Blood samples were collected from 350 dairy cattle on 5 farms in Guangzhou City, southern China between July 2009 and January 2010. The dairy cattle populations represented a local breed (Chinese Holstein) and an introduced breed (American/Australian Holstein-Friesian and British Jersey). All the blood samples were immediately transported to the laboratory at The College of Veterinary Medicine, South China Agricultural University. Serum was separated by centrifugation at 3,000 rpm for 10 min. The serum samples were stored at -20°C until tested for antibodies against *T. gondii*. Biometric data for dairy cattle, includingage, breed and numbers of past pregnancies were obtained from the farmers.

### Serological examination

Antibodies against *T. gondii *were determined by IHA using a commercially-marketed kit (Veterinary Research Institute, Jiangsu Academy of Agricultural Sciences, Jiang Province, China) according to the manufacturer's protocol as described previously [14,15]. The test was considered positive if a layer of agglutinated erythrocytes was formed in wells at serum dilutions of 1:64 or higher. Positive and negative control sera were provided in the kit and were included in each test. The positive control sera were collected from pigs experimentally infected with *T. gondii*. The negative control sera were collected from pigs without *T. gondii *infection (collected before experimental infection).

### Statistical analysis

Differences in *T. gondii *seroprevalence among dairy cattle of different age groups and different numbers of pregnancies were analyzed by a Chi square test using the SPSS for Windows (Release 18.0 standard version, SPSS Inc., Chicago, Illinois). The differences were considered statistically significant when *P *< 0.05.

## Results

A total of 350 dairy cattle from 5 farms in Guangzhou, Southern China were examined by IHA for *T. gondii *antibodies. 20 of the 350 (5.7%) examined dairy cattle were seropositive for *T. gondii *infection by IHA at the cut-off of 1:64 (Table 1). Different levels of *T. gondii *seropositivity were detected in 5 different farms (Table 1). Seroprevalence varied in different age groups, ranging from 2.3% to 12.5% (Table 2). The numbers of parturition of dairy cattle ranged between 1 pregnancy and 7 pregnancies, and the *T. gondii *seroprevalence varied in dairy cattle with different numbers of pregnancies, ranging from 0 to 11.5% (Table 3).

**Table 1:** Seroprevalence of *Toxoplasma gondii *infection in dairy cattle in Guangzhou, southern China

Farm	No. examined	No. positive	Prevalence (%)
A	80	2	2.5

B	50	3	6

C	60	4	6.7

D	80	4	5

E	80	7	8.8

Total	350	20	5.7

**Table 2:** Seroprevalence of *Toxoplamsa gondii *in different ages of dairy cattle in Guangzhou, southern China

Age (year)	No. examined	No. positive	Prevalence (%)
1 ≤ yr < 2	44	1	2.3

2 ≤ yr < 3	51	2	3.9

3 ≤ yr < 4	54	2	3.7

4 ≤ yr < 5	25	2	8

5 ≤ yr < 6	48	6	12.5

6 ≤ yr < 7	42	3	7.1

7 ≤ yr < 8	57	2	3.5

≥ 8	29	2	6.9

Total	350	20	5.7

**Table 3:** Seroprevalence of *Toxoplasma gondii *in dairy cattle of different pregnancies in Guangzhou, southern China

No. pregnancies	No. examined	No. positive	Prevalence (%)
0	48	1	2.1

1	35	3	8.6

2	41	2	4.9

3	52	6	11.5

4	39	3	7.7

5	48	2	4.2

6	44	2	4.5

≥ 7	17	0	0

With no history records	26	1	3.8

Total	350	20	5.7

## Discussion

In this study, we examined the seroprevalence of *T. gondii *infection in dairy cattle in Guangzhou city, southern China. 5.7% of the 350 tested dairy cattle were seropositive for *T*. *gondii *by IHA, which is lower than that reported in Guangxi (9.2%), Liaoning (6.0%), Qinghai (11.8%), Xinjiang (46.4%) in China [16-19] and some other countries [20-25], but higher than that reported in Yunnan province (1.4%) [26]. Different *T. gondii *seroprevalences in dairy cattle in different countries and regions may be due to different serological tests used and different sources of dairy cattle. *T. gondii *infection is probably more prevalent in warm and humid areas than in cold and dry regions [1]. This is probably related to conditions relating to the survival of oocysts in the environment.

The ages of the 350 dairy cattle were analyzed for the association with *T. gondii *seroprevalence (Table 2). The seroprevalence varied in different age groups, ranging from 2.3%-12.5%, with the highest of 12.5% in dairy cattle which were < 6 year old or ≥ 5 year old, although the seroprevalences were not statistically significantly different among the different age groups (*P *> 0.05). The varied seroprevalence in different age groups suggests the possibility of horizontal transmission in the investigated herds.

Of the 350 dairy cattle examined, 322 had record of numbers of previous pregnancies for the analysis of the association between *T. gondii *seroprevalence and past pregnancies (Table 3). The seroprevalence in the dairy cattle with 3 births was the highest (11.5%), followed by the dairy cattle with 1 pregnancy, but the differences were not statistically significant (*P >*0.05).

## Conclusions

This survey demonstrated that *T. gondii *infection is prevalent in dairy cattle of all age ranges in Guangzhou, China, which may represent a potential source for human infection with *T*. *gondii*. Therefore, integrated strategies and measures should be executed to control and prevent *T. gondii *infection in dairy cattle in the study region.

## Competing interests

The authors declare that they have no competing interests.

## Authors' contributions

XQZ and SJL conceived and designed the study, and critically revised the manuscript. DHZ, FRZ and HYX performed the experiments, analysed the data and drafted the manuscript. PL, MJX, LGY, CY and SYH helped in study design, study implementation and manuscript revision. All authors read and approved the final manuscript.
